# The Communication Between the Immune and Nervous Systems: The Role of IL-1β in Synaptopathies

**DOI:** 10.3389/fnmol.2018.00111

**Published:** 2018-04-05

**Authors:** Davide Pozzi, Elisabetta Menna, Alice Canzi, Genni Desiato, Cristina Mantovani, Michela Matteoli

**Affiliations:** ^1^Department of Biomedical Sciences, Humanitas University, Rozzano, Italy; ^2^Humanitas Clinical and Research Center, Rozzano, Italy; ^3^Istituto di Neuroscienze, Consiglio Nazionale delle Ricerche, Milan, Italy; ^4^School of Medicine and Surgery, University of Milan-Bicocca, Milan, Italy

**Keywords:** synaptopathies, inflammation, cytokines, IL-1β, IL1RAPL1, neurodevelopmental diseases, KCC2, MeCP2

## Abstract

In the last 15 years, groundbreaking genetic progress has underlined a convergence onto coherent synaptic pathways for most psychiatric and neurodevelopmental disorders, which are now collectively called “synaptopathies.” However, the modest size of inheritance detected so far indicates a multifactorial etiology for these disorders, underlining the key contribution of environmental effects to them. Inflammation is known to influence the risk and/or severity of a variety of synaptopathies. In particular, pro-inflammatory cytokines, produced and released in the brain by activated astrocytes and microglia, may play a pivotal role in these pathologies. Although the link between immune system activation and defects in cognitive processes is nowadays clearly established, the knowledge of the molecular mechanisms by which inflammatory mediators specifically hit synaptic components implicated in synaptopathies is still in its infancy. This review summarizes recent evidence showing that the pro-inflammatory cytokine interleukin-1β (IL-1β) specifically targets synaptopathy molecular substrate, leading to memory defects and pathological processes. In particular, we describe three specific pathways through which IL-1β affects (1) synaptic maintenance/dendritic complexity, (2) spine morphology, and (3) the excitatory/inhibitory balance. We coin the term immune synaptopathies to identify this class of diseases.

## Introduction

A large amount of evidence about the genetic architecture of psychiatric and neurodevelopmental diseases has progressively accumulated in the last 15 years or so. The identified pathways typically involve proteins chiefly affecting synapse formation and maintenance, a discovery which has led to the concept of “synaptopathies” (Grant, 2012). The initial excitement raised by these findings has been, however, tempered by the awareness that understanding a given synaptopathy at the level of its genetic, molecular, and synaptic dysfunction is typically insufficient to explain the disease onset, which depends indeed on additional genetic, epigenetic, and environmental factors (Beutner et al., 2007; Oh-Nishi et al., 2010; De Chiara et al., 2012; Millan, 2013; Giovanoli et al., 2016).

Inflammation is increasingly recognized as a key factor influencing physiology and pathology in the immature and mature brain, which can be exposed to inflammation in connection with viral or bacterial prenatal or postnatal infections or as a result of sterile CNS insults (Hagberg et al., 2015). Extensive research is providing evidence that inflammation has long-term consequences and could speculatively affect the risk and/or severity of a variety of brain diseases, including autism spectrum disorders (ASDs), schizophrenia, and intellectual disabilities (IDs), which represent recognized synaptopathies (Fan et al., 2007; Dantzer et al., 2008; Najjar et al., 2013). Accordingly, prenatal and early postnatal infections have been associated with increased risk for a number of neurodevelopmental disorders (Brown and Derkits, 2010; Hagberg et al., 2012; Lipina et al., 2013; Wischhof et al., 2015; Graham et al., 2018).

Pro-inflammatory cytokines, including interleukin-1β (IL-1β), interleukin-6 (IL-6), and tumor necrosis factor alpha (TNFα), appear to be at the forefront in the communication between the immune and the nervous system, playing dual roles in mediating physiological and neuroprotective roles in normal brain function (Kushima et al., 1992; Yamada and Hatanaka, 1994; Akaneya et al., 1995; Hirota et al., 1996; Wagner, 1996; Gadient and Otten, 1997; Parish et al., 2002; Nakanishi et al., 2007; Heese, 2017) or being detrimental and associated with brain diseases, especially when present at elevated concentrations (Yan et al., 1992; Katila et al., 1994; Gadient and Otten, 1997; Samuelsson et al., 2006; Garay and McAllister, 2010; Ashwood et al., 2011; Suzuki et al., 2011; Wei et al., 2011; Erta et al., 2012; Chase et al., 2016; O’Keeffe, 2017). In particular, experiments performed in rodents have unequivocally demonstrated that inflammation correlates with defective learning and memory paradigms. As an example, influenza infection associated to elevated pro-inflammatory cytokines was found to alter neuronal morphology leading to cognitive impairment in adult mice (Jurgens et al., 2012; Hosseini et al., 2018). Although the link between immune system activation and defects in cognition is solidly established, the molecular underpinnings of this correlation are not completely clear. In particular, the knowledge of whether inflammatory mediators specifically hit synaptic components, previously identified by genetic studies as implicated in synaptopathies, is poor and somehow fragmentary.

## Excessive IL-1β Impairs Neuronal Plasticity and Memory

Interleukin-1β is a potent inflammatory cytokine and a fundamental component of the innate immune response (Dinarello, 1996). Besides affecting several organs during inflammatory processes, IL-1β also exerts a number of diverse actions in the central nervous system (CNS) as important mediator of neuronal injury. Twenty years of research have indeed indicated that IL-1β is involved in several brain diseases, including multiple sclerosis (Lin and Edelson, 2017), Alzheimer disease (Griffin et al., 2006), epilepsy (Iori et al., 2016), stroke (Murray et al., 2015), and even neurodevelopmental disorders such as schizophrenia and autism (Soderlund et al., 2009; Girard et al., 2010; Krakowiak et al., 2017).

Despite the heterogeneity of the diseases in which IL-1β is involved, a growing body of evidence points toward a shared physiological process hit by the cytokine: cognition. This concept originally emerged as the result of a series of pioneer experiments showing that the intraperitoneal or intrahippocampal injection of the cytokine results in learning and memory defects (Oitzl et al., 1993; Gibertini et al., 1995; Barrientos et al., 2002). The detrimental effects of IL-1β on cognition were later confirmed using transgenic mice expressing the cytokine in an inducible manner (Hein et al., 2010) or upon endogenous IL-1β elevations evoked by infections (Gibertini et al., 1995; Barrientos et al., 2006; Chen et al., 2008), with the memory deficits being prevented, in the latter case, by intraventricular infusion of the naturally occurring interleukin-1 receptor antagonist (IL-1ra; Goshen et al., 2007; Barrientos et al., 2009; Frank et al., 2010).

In line with the induction of cognitive defects, excessive IL-1β affects long-term potentiation (LTP), the synaptic process which underlies learning and memory. Indeed, elevated levels of IL-1β inhibit LTP in several regions of the hippocampus, including CA1 (Bellinger et al., 1993; Ross et al., 2003), CA3 (Katsuki et al., 1990), and dentate gyrus (Murray and Lynch, 1998; Kelly et al., 2003). Of note, the synaptic potentiation processes are not only affected in different pathological scenarios, but also during aging, when the overproduction of IL-1β and/or a synapse-specific IL-1 receptor subunit reconfiguration may produce specific deficits in consolidation of hippocampus-dependent memory (Patterson, 2015; Prieto et al., 2015) and also during stress-related conditions, a particular pathophysiological state which might deeply affect immature brain (Barron et al., 2017; Depino, 2017; Schiavone and Trabace, 2017).

## IL-1β Affects Synapse Structure and Function

An extensive series of evidence accumulated over the last 10 years has pointed to the concept that IL-1β directly affects synapse structure and function. Indeed, early experiments performed in primary cultured neurons exposed to recombinant IL-1β revealed a significant decline in the levels of the synaptic vesicle protein synaptophysin (Li et al., 2003) and in the number of synaptic sites (Mishra et al., 2012). The occurrence of deleterious cytokine effects on the synapse structure has been reported in different non-neurological disorders associated to increases of IL-1β levels, like sepsis and obesity, where mice show memory impairment and reduced number of hippocampal and cortical excitatory synapses, through a mechanism fully prevented by the addition of IL-1ra (Erion et al., 2014; Moraes et al., 2015).

Different mechanisms have been called into question to shed light on the association between excessive IL-1β and synaptic alterations, including the modulation of the mitogen-activated protein kinase (MAPK) pathway and the modification of trophic factor production, such as brain-derived neurotrophic factor (BDNF). These processes have been thoroughly analyzed in a previous review (Patterson, 2015). Conversely, it is still unclear whether IL-1β may directly act on neuronal proteins involved in modulating the structure and function of dendritic spines and known to be the molecular targets of synaptopathies. In this review, we will illustrate some recently reported data showing that IL-1β may directly interfere with synaptic processes known to be at the root of neurodevelopmental diseases. The research was performed through PubMed. Inclusion criteria were based on the indicated keywords. Only peer-reviewed original articles and reviews were considered.

## IL1RAPL1 and Synapse Stabilization

Interleukin-1-receptor accessory protein like 1 (IL1RAPL1) is a member of the interleukin-1 receptor family. It is selectively expressed in the brain (Carrie et al., 1999; Born et al., 2000), where it is mainly localized at excitatory synapses. Mutations in the gene encoding for IL1RAPL1 have been found in patients with cognitive impairments ranging from non-syndromic ID to ASD (Ramos-Brossier et al., 2015). Indeed, IL1RAPL1 KO mice display a reduction of spine density in the cortex (Yasumura et al., 2014) and in the CA1 region of the hippocampus (Pavlowsky et al., 2010; Yasumura et al., 2014) and are characterized by altered excitation/inhibition (E/I) balance in the cerebellum and amygdala (Gambino et al., 2009; Houbaert et al., 2013). These molecular and functional alterations are associated with memory deficits (Houbaert et al., 2013; Yasumura et al., 2014). In neurons, IL1RAPL1 plays a role in presynaptic differentiation, in spine formation and stabilization (Pavlowsky et al., 2010; Valnegri et al., 2011; Yoshida et al., 2011; Ramos-Brossier et al., 2015), and in dendritic morphology (Montani et al., 2017). Also IL1RAPL1 C-terminus interacts with the neuronal calcium sensor-1 which regulates voltage-gated calcium channel activity (Gambino et al., 2009). Thus, IL1RAPL1 may promote excitatory synapse formation through two main mechanisms: (i) a trans-synaptic signaling pathway involving the receptor tyrosine phosphatase δ (PTPδ) and RhoGAP2 (Valnegri et al., 2011) and (ii) the control of synaptic localization of PSD-95 through c-Jun N-terminal kinase (JNK) activity and PSD-95 phosphorylation (Pavlowsky et al., 2010).

**FIGURE 1:**
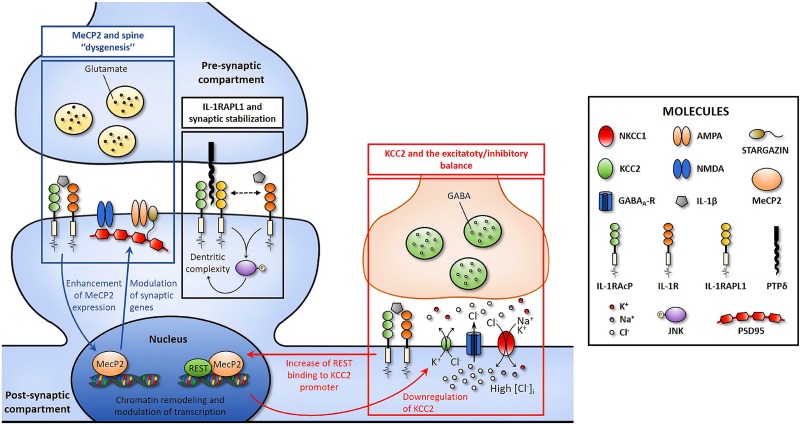
Cartoon depicting three possible pathways through which IL-1β might affect synaptic function and structure by acting on IL-R1 which is localized at synaptic site (Gardoni et al., 2011). *Pathway 1*: IL1RAPL1 mediates some of the effects of IL-1β in neurons, altering dendritic morphology. *Pathway 2*: IL-1β affects spine morphogenesis enhancing the expression of the transcription factor Mecp2, which in turn alters the expression of synaptic proteins via chromatin-remodeling mechanisms. *Pathway3*: IL-1β modulates the excitatory to inhibitory switch of GABA by reducing the expression of the chloride transporter KCC2, leading to E/I unbalance and higher susceptibility to seizures. Pathways 2 and 3 could be linked based on the REST/MeCP2 ability to bind KCC2 gene promoter and act as a repressor of the transporter transcription (see text for details).

IL1RAPL1 mediates some of the effects of IL-1β in neurons (**Figure 1**, pathway 1), controlling, in particular, the cytokine effects on dendritic morphology, possibly through the involvement of JNK pathway (Pavlowsky et al., 2010; Montani et al., 2017). IL1RAPL1 represents therefore the first identified synaptic target of IL-1β. The primary role of the protein in neuronal and synaptic development, together with the observation that IL1RAPL1 is a synaptopathy-related gene, identifies this protein as one of the possible key targets of the immune-to-neuron communication mediated by IL-1β. The future identification of the precise mechanisms by which IL1RAPL1 modulates the activity of IL-1β could offer the opportunity to specifically interfere with the vicious, harmful cycle leading to synapse dysfunction.

## MeCp2 and Spine “Dysgenesis”

Recent studies have exploited a genetic mouse model of IL-1β deregulation, the IL-1R8 KO mice, as a reliable and reproducible system for examining the effects of inflammation on synapse structure and function, also elucidating the molecular processes involved (Costello et al., 2011; Tomasoni et al., 2017). IL-1R8, also known as single Ig IL-1 related receptor (SIGIRR), reduces the activation of the Toll-like receptors (TLRs; protein sensors for microorganisms and tissue damage) and IL1R signaling pathways by intracellularly interfering with the association of adaptor molecules to the receptor complex including nuclear factor kappa-light-chain-enhancer of activated B cells (NF-κB) and JNK, thus leading to upregulation of IL-1 signaling. Mice lacking IL-1R8, previously shown to exhibit LTP deficits (Costello et al., 2011), have been used to investigate the mechanisms through which exaggerated inflammatory conditions impact synapse functions. IL-1R8KO neurons were found to display an increased number of immature, thin spines, and a decreased number of mature, mushroom spines, accompanied by reduction of PSD95 expression and impairment of synaptic plasticity (Tomasoni et al., 2017). The phenotype was rescued by IL-1ra, thus proving the direct involvement of IL-1β signaling (Tomasoni et al., 2017). The structural and functional alterations were found to be causally linked to upregulation of the mammalian target of rapamycin (mTOR) pathway and increased levels of the epigenetic regulator methyl CpG binding protein 2 (MeCP2), which were again normalized by IL-1Ra treatment (Tomasoni et al., 2017). The demonstration that enhanced IL-1β signaling increases the expression of MeCP2, thus negatively impacting synapse function, points to this factor as a potentially fundamental node linking inflammation and synaptic damage (**Figure 1**, pathway 2). Of note, alterations in the expression of MeCP2 are known to be responsible of neurodevelopmental diseases in humans: sporadic, loss-of-function mutations in the gene coding for MeCP2 result in Rett syndrome (Amir et al., 1999), while a double dosage of MeCP2 causes a severe developmental delay and ID, with even mild over-expression having a robust effect (Delobel et al., 1998; Van Esch et al., 2005). Interestingly, IL-1Ra reduces MeCP2 levels in wild-type neurons which, concomitantly, lose the ability to undergo LTP (Tomasoni et al., 2017). It appears therefore that the effects of IL-1β on neuronal plasticity follow a U-shaped dose–response curve, with levels in either excess or below the physiological range being deleterious to neuronal functions, which is exactly the case of MeCP2, whose levels need to be tightly regulated to guarantee a proper neuronal function (Cheng and Qiu, 2014; Lombardi et al., 2015). It will be important to assess whether the IL-1β-mediated modulation of MeCP2 also occurs in humans and to define the molecular mechanisms by which the cytokine modulates MeCP2 levels.

## KCC2 and the Excitatory/Inhibitory Balance

Several lines of evidence have highlighted the capacity of IL-1β to promote an imbalance between excitation and inhibition, thus affecting neuronal network excitability. The first indications were obtained in the peripheral nervous system, where elevated levels of pro-inflammatory cytokines, including IL-1β, were found to alter neuronal excitability facilitating pain and hyperalgesia (Viviani et al., 2007; Schafers and Sorkin, 2008). Further studies conducted on the CNS have subsequently demonstrated that high levels of IL-1β, as occurring in neurological disorders such as multiple sclerosis and epilepsy, lead to an E/I imbalance, which might be responsible for – or contribute to – cognitive impairment (Vezzani et al., 2008; Rossi et al., 2012; Iori et al., 2016). Among the possible mechanisms, neuronal network hyperactivity may be the result of an altered excitatory-to-inhibitory switch of GABA signaling, leading to a reduced inhibitory action of GABA (Ganguly et al., 2001; Tyzio et al., 2007; Ben-Ari et al., 2012b), a process tightly controlled by the developmentally regulated expression of the two chloride co-transporters, potassium-chloride cotransporter 2 (KCC2) and neuronal Na–K–Cl cotransporter 1 (nKCC1) (Ben-Ari et al., 2012a; Watanabe and Fukuda, 2015; Raimondo et al., 2017). This evidence raised the question of whether an inflammatory event occurring during brain development may specifically affect this process. Such possibility has been called into question in a recent study, where an immune challenge – consisting in the injection of the viral mimicking molecule polyinosinic–polycytidylic acid (poly I:C) – was delivered to pregnant mice at early stages of embryo development (E9, corresponding to the mid/end of first trimester pregnancy in humans), a model of prenatal infection called maternal immune activation (MIA). The prenatal immune activation resulted in a delay of GABA switch in the offspring, resulting from a higher cortical and hippocampal expression of the transporter nKCC1 and a lower expression of KCC2 compared to controls. The alterations in nKCC1/KCC2 ratio resulted in GABA being excitatory and offspring more susceptible to seizures in the adult stage. The involvement of IL-1β in the process was indicated by the evidence that (i) IL1RKO embryos are protected from this detrimental effect and (ii) IL-1β delays the transition of GABA signaling in cultured neurons (**Figure 1**, pathway 3; Corradini et al., 2017). Among the transcriptional regulators of KCC2 expression, the neuronal repressor gene RE1-silencing transcription factor (REST) was found to modulate *kcc2* gene expression (Yeo et al., 2009) and, in line with this evidence, its binding to KCC2 promoter was enhanced in the brain of offspring exposed to MIA (Corradini et al., 2017). However, given that MeCP2 also acts as a transcriptional repressor by binding to the KCC2 gene promoter (Tang et al., 2016), it will be interesting to define the interplay between these two factors and whether the increase of MeCP2 levels induced by IL-1β (Tomasoni et al., 2017) might contribute to KCC2 reduction.

The demonstration that a transient increase of IL-1β during neuronal development may induce long-lasting neuronal network hyperactivity by delaying GABA switch opens unexpected scenarios: impairment of the GABA developmental switch is indeed an important feature of several neurodevelopmental disorders, such as Down syndrome, Rett syndrome, and ASD (Lemonnier et al., 2012; He et al., 2014; Tyzio et al., 2014; Deidda et al., 2015; Inui et al., 2017), for which an important risk factor is represented by prenatal inflammatory conditions (Tarnow-Mordi et al., 2005; Missault et al., 2014).

An E/I imbalance has also been demonstrated in other neurological diseases characterized by excessive IL-1β levels, such as multiple sclerosis and epilepsy. In these cases, alternative molecular mechanisms were found to be involved. In a mouse model of multiple sclerosis, IL-1β-mediated inflammation was found to enhance glutamatergic transmission during the early phase of the disease (Centonze et al., 2009; Rossi et al., 2012, 2014; Mandolesi et al., 2013), through modulating vanilloid 1 channels in hippocampus (Rossi et al., 2012), downregulating the glutamate–aspartate transporter/excitatory amino acid transporter 1 (GLAST/EAAT1) in the cerebellum (Mandolesi et al., 2013, 2017), and by activating the apoptotic cascade through p53 activation (Rossi et al., 2014). An IL-1β-dependent enhancement of excitatory neurotransmission has also been reported in epilepsy (Iori et al., 2013) where danger signals such as High Mobility Group Box 1 (HMGB1) and Toll-like receptor 4 (TLR4) were proposed to mediate the IL-1β-dependent increase in neuronal excitability, through an Src kinase-mediated phosphorylation of the NR2B subunit of the *N*-methyl-D-aspartate (NMDA) receptor (Viviani et al., 2003; Maroso et al., 2010, 2011). Together these data indicate that IL-1β disrupts the correct E/I equilibrium acting on multiple molecular targets.

## Conclusion and Perspectives

**FIGURE 2:**
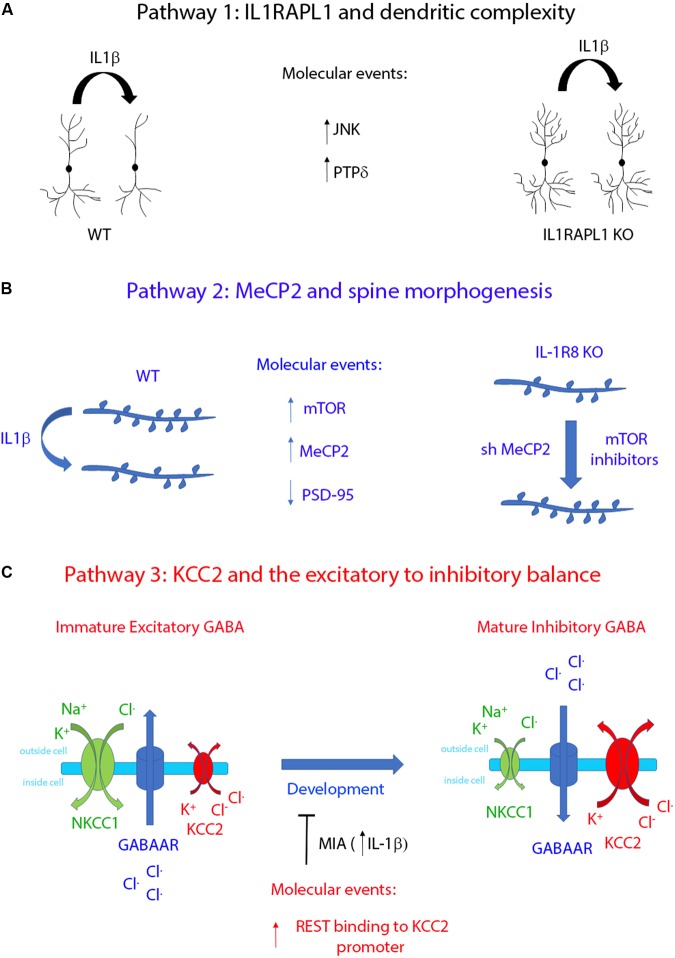
Schematic representation summarizing the functional effects of the three regulatory pathways controlled by IL-1β. **(A)** IL-1β affects dendritic complexity through IL1RAPL1-dependent mechanisms. This pathway activates JNK and PTPδ intracellular proteins (Montani et al., 2017). **(B)** IL-1β regulates dendritic spine morphology by upregulating the transcription factor MeCP2 in an mTOR-dependent manner (Tomasoni et al., 2017). **(C)** IL-1β leads to an excitatory/inhibitory unbalance by delaying the developmentally regulated switch of GABA signaling. This pathway involves the interplay between the two transcription factors REST and MeCP2, which in turn regulates the transcriptional level of KCC2 (Corradini et al., 2017).

It is now clear that inflammation is an important contributor to defects in brain function, by affecting in particular cognitive processes (Rachal Pugh et al., 2001; Huang and Sheng, 2010; Yirmiya and Goshen, 2011) through concentration-dependent harmful effects induced by the pro-inflammatory cytokine IL-1β. Indeed, recent reports have highlighted the ability of IL-1β to selectively affect cell-to-cell communication in the brain, by targeting specific synaptic pathways (Mishra et al., 2012; Han et al., 2017) which are known to be altered in different synaptopathies (see **Figures 1**, **2**). Among these, IL1RAPL1, MeCP2, and KCC2 are three central molecular players of neurodevelopmental disorders, whose functionality may be affected by IL-1β.

These results may have important translational implications. As an example, the recognition that IL-1β modulates MeCP2 levels opens the challenging possibility to target the immune system for treating neurodevelopmental diseases characterized by altered levels of this transcription factor. Observational clinical data already support the possibility to treat cognitive symptoms by immunomodulatory drugs. Indeed, in patients affected by cryopyrin-associated periodic syndrome (CAPS), a group of rare genetic autoinflammatory diseases with levels of IL-1β being fivefold higher than in healthy individuals, symptoms of ID frequently occur. These cognitive defects are reversed following treatment with IL-1Ra (Bachove and Chang, 2014) or with specific neutralization of IL-1β with canakinumab (Kuemmerle-Deschner et al., 2011).

Although the modulation of IL-1β signaling in neurology or psychiatry is still in its infancy, the possibility of selectively inhibiting specific steps in the IL-1β cascade may represent a unique opportunity to treat immune synaptopathies (**Box 1**), i.e., synaptic dysfunctions resulting from deregulation of the immune system during brain development. A control of maternal inflammatory parameters during pregnancy may turn out to be a promising strategy to decrease the incidence of immune-mediated neurological or psychiatric illness in adulthood, as a consequence of maternal viral infections. Specific studies aimed at testing the efficacy of different IL-1β blockers that offer potential benefits to patients in individual disease states will be required in the next future.

**Box 1. Synaptopathies and inflammation: the concept of immune-synaptopathies.**

The term synaptopathy (from Greek συν, syn – together, απτειν, haptein – to clasp, and παθοϛ, pathos) refers to a class of neurological disorders characterized by alterations at the synaptic level (Brose et al., 2010). The concept of synaptopathy is based on several genetical studies which have identified the involvement, in different diseases, of specific genes whose products converge onto coherent biological pathways controlling various aspects of synaptic structure and function (see **Table 1**). Besides neurodevelopmental diseases, other brain diseases have been identified as synaptopathies, including neurodegenerative disorders, such as Alzheimer’s (Kerrigan and Randall, 2013) and Parkinson’s (Longhena et al., 2017) diseases. Synaptopathies are therefore defined as resulting from pathological events including synaptic loss (in which synaptic connectivity is compromised), alterations of synaptic functioning (in which the physiological activity of synapses is altered), or both (for a review, see Lepeta et al., 2016). We introduce the novel term immune-synaptopathy to underline the concept that the activation of the immune system, resulting in the formation of soluble immune mediators (i.e., cytokines), which are the main key effectors of the inflammatory response, directly impacts the physiological activity of the synapse producing a disease state (see text for details).

**Table 1:** List of selected synaptic and/or synaptic related gene disease.

Protein	Gene	Function	Disease (selected)	Reference
Complexin-1	Cplx1	Transmitter release	HD, SCZ	Brose, 2008; but also Kishi et al., 2006
Complexin-2	Cplx2	Transmitter release	HD, SCZ	Brose, 2008; but also Kishi et al., 2006
Munc 18-1	Stxbp1	Transmitter release	AD, EPI, PD	Jacobs et al., 2006; Saitsu et al., 2008; Chai et al., 2016
SNAP-25	SNAP25	Pre and postsynaptic function	ADHD, SCZ, BP, AD	Thompson et al., 1998; Kustanovich et al., 2003; Thapar et al., 2007; Guerini et al., 2014; Antonucci et al., 2016; Houenou et al., 2017; Kang et al., 2017
Neurexin-1	Nrxn1	Synaptogenesis	ASD, SCZ	Kim et al., 2008; Sudhof, 2008; Vaags et al., 2012
Neuroligin-3	Nlgn3	Synaptogenesis	ASD	Jamain et al., 2003; Sudhof, 2008; Burrows et al., 2015
Neuroligin-4	Nlgn4	Synaptogenesis	ASD, ID, TOU	Jamain et al., 2003; Lawson-Yuen et al., 2008; Sudhof, 2008
PSD-95	Dlg4	Postsynaptic function	SCZ, ASD	Toro and Deakin, 2005; Kristiansen et al., 2006; Xing et al., 2016
PSD-93	Dlg2	Postsynaptic function	SCZ, ASD	Kristiansen et al., 2006; Egger et al., 2014
SAP-102	Dlg3	Postsynaptic function	Xlinked ID, SCZ	Oldmeadow et al., 2014; Tzschach et al., 2015; Gieldon et al., 2017
GLUN2B	Grin2B	Receptor function	SCZ, ASD, DEP	Toro and Deakin, 2005; Kristiansen et al., 2006; Zhang et al., 2014
SHANK-3	Shank3	Postsynaptic function	ASD	Durand et al., 2007
DISC1	DISC1	Synaptogenesis	SCZ	Hayashi-Takagi and Sawa, 2010; Hayashi-Takagi et al., 2010
TNIK	TNiK	Postsynaptic function	SCZ	Coba et al., 2012
C4 (complement component 4)	C4	Synaptic pruning	SCZ	Sekar et al., 2016
MeCP2	MeCP2	Regulation of gene expression	Rett	Amir et al., 1999
FMRP	FMR1	Synaptogenesis	FRAXA, ID, ASD	Hagerman and Hagerman, 2004; Hagerman et al., 2004
Gephyrin	GPHN	Receptor function	ASD, EPI	Egger et al., 2014; Dejanovic et al., 2015


*AD, Alzheimer’s disease; ADHD, attention deficit hyperactivity disorder; ASD, autism spectrum disorder; BP, bipolar disorder; DEP, depression; EPI, epilepsy; FRAXA, fragile X syndrome; HD, Huntington’s disease; ID, intellectual disability; SCZ, schizophrenia; TOU, Tourette syndrome; Rett, Rett syndrome; X-link ID, X-linked intellectual disability.*

## Author Contributions

DP, EM, and MM designed the review outline. All the authors contributed to writing and designing the scheme. CM realized the figure.

## Conflict of Interest Statement

The authors declare that the research was conducted in the absence of any commercial or financial relationships that could be construed as a potential conflict of interest.
